# Cell wall proteome of sugarcane stems: comparison of a destructive and a non-destructive extraction method showed differences in glycoside hydrolases and peroxidases

**DOI:** 10.1186/s12870-015-0677-0

**Published:** 2016-01-11

**Authors:** Maria Juliana Calderan-Rodrigues, Elisabeth Jamet, Thibaut Douché, Maria Beatriz Rodrigues Bonassi, Thaís Regiani Cataldi, Juliana Guimarães Fonseca, Hélène San Clemente, Rafael Pont-Lezica, Carlos Alberto Labate

**Affiliations:** Departamento de Genética, Laboratório Max Feffer de Genética de Plantas, Escola Superior de Agricultura “Luiz de Queiroz”, Universidade de São Paulo, Av. Pádua Dias 11, CP 83, 13400-970 Piracicaba, SP Brazil; Université de Toulouse; UPS; UMR 5546, Laboratoire de Recherche en Sciences Végétales, BP 42617, F-31326 Castanet-Tolosan, France; CNRS; UMR 5546, BP 42617, F-31326 Castanet-Tolosan, France

**Keywords:** Cell wall protein, *Saccharum* sp, Stem, Proteomics, Second generation ethanol

## Abstract

**Background:**

Sugarcane has been used as the main crop for ethanol production for more than 40 years in Brazil. Recently, the production of bioethanol from bagasse and straw, also called second generation (2G) ethanol, became a reality with the first commercial plants started in the USA and Brazil. However, the industrial processes still need to be improved to generate a low cost fuel. One possibility is the remodeling of cell walls, by means of genetic improvement or transgenesis, in order to make the bagasse more accessible to hydrolytic enzymes. We aimed at characterizing the cell wall proteome of young sugarcane culms, to identify proteins involved in cell wall biogenesis. Proteins were extracted from the cell walls of 2-month-old culms using two protocols, non-destructive by vacuum infiltration *vs* destructive. The proteins were identified by mass spectrometry and bioinformatics.

**Results:**

A predicted signal peptide was found in 84 different proteins, called cell wall proteins (CWPs). As expected, the non-destructive method showed a lower percentage of proteins predicted to be intracellular than the destructive one (33 % *vs* 44 %). About 19 % of CWPs were identified with both methods, whilst the infiltration protocol could lead to the identification of 75 % more CWPs. In both cases, the most populated protein functional classes were those of proteins related to lipid metabolism and oxido-reductases. Curiously, a single glycoside hydrolase (GH) was identified using the non-destructive method whereas 10 GHs were found with the destructive one. Quantitative data analysis allowed the identification of the most abundant proteins.

**Conclusions:**

The results highlighted the importance of using different protocols to extract proteins from cell walls to expand the coverage of the cell wall proteome. Ten GHs were indicated as possible targets for further studies in order to obtain cell walls less recalcitrant to deconstruction. Therefore, this work contributed to two goals: enlarge the coverage of the sugarcane cell wall proteome, and provide target proteins that could be used in future research to facilitate 2G ethanol production.

**Electronic supplementary material:**

The online version of this article (doi:10.1186/s12870-015-0677-0) contains supplementary material, which is available to authorized users.

## Background

The use of *Saccharum* sp. to produce second generation (2G) ethanol can reduce waste and increase the yield without expanding the crop area, contributing to a cleaner, more efficient and more sustainable production. However, from the economic point of view, the costs of the process need to be reduced, mostly those related to the enzymes used to deconstruct plant cell walls. Therewith, research is mainly focused on the identification of new enzymes that could efficiently degrade cell walls [1]. Other studies have been developed from the biomass perspective, describing the plant cell wall components [2–5], and even altering them attempting to achieve a higher ethanol 2G yield. Since pre-treatments facilitate cell wall digestibility to increase ethanol production, when altering plant cell wall components, focus should be either on lignin- carbohydrate complex cleavage and hemicellulose removal, or lignin modification and even on redistribution and cellulose decrystallization [6].

Plant cell walls are mainly composed of polysaccharides and cell wall proteins (CWPs) [7]. Proteomics studies have revealed the large diversity of CWPs [8–10]. They have been grouped in different functional classes according to predicted functional domains and experimental data: polysaccharide modifying proteins, oxido-reductases and proteases, have been found as major classes. Structural proteins such as hydroxyproline-rich glycoproteins, namely extensins, arabinogalactan proteins and hydroxyproline/proline-rich proteins, have been estimated to account for about 10 % of the cell wall mass in dicots [11] and approximately 1 % in monocots [12]. However, only a few of them have been identified in proteomics studies. CWPs are involved in growth and development, signaling and defense against pathogens. They virtually take part in most functions of the cells [4, 11, 13]. They can affect cell fate, being able to sense stress signals and transmitting them to the cell interior [14]. They can also have tissue-specific functions , such as playing roles in cuticle formation [15]. Due to this versatility, plant cell walls are the subject of many fields of research.

In the case of grasses, type II-cell walls present specific features [7]. The cellulose microfibrils are interlocked by glucuronoarabinoxylans, instead of xyloglucans of type I-cell walls. In addition, the grass cell walls contain a substantial portion of non-cellulosic polymers ‘wired on’ the microfibrils by alkali-resistant phenolic linkages.

As mentioned above, plant cell walls contain enzymes capable of modifying the cell wall matrix [16]: endoglucanases which cleave the polysaccharide backbones; glycosidases which remove side chains; transglycosylases which cut the polysaccharides and link them together; esterases which remove methyl groups of pectins, and cleave ester bonds in polysaccharide chains; and class III peroxidases (Prxs) which form or break phenolic bonds. Altogether, these enzymes offer many possibilities to modify the structure and the mechanical properties of cell walls, and thus biomass structure [3]. Besides, the addition of plant glycosidases during the hydrolysis of corn stover could increase the ethanol yield [17]. These examples show that the repertoire of CWPs could provide interesting tools to improve the deconstruction of cell walls.

As commonly known, classical CWPs share common features. The first one is a signal peptide at the *N*-terminus of the protein which is responsible for their targeting to the endoplasmic reticulum (ER) [18], the first organelle of the secretory pathway [19]. The signal peptide is not formed by a consensus amino acid sequence. However, it has a positively charged n-region at its N-terminus and a central hydrophobic h-region followed by a polar c-region at its C-terminus comprising the cleavage site [20]. In addition, CWPs do not possess the canonical ER retention signal KDEL or HDEL tetrapeptide at their C-terminus [19, 20]. The third feature is that they do not present a trans-membrane domain. When passing through the secretory pathway, proteins go from ER to the Golgi complex in order to be packed into vesicles and directed to be secreted. Plasma membrane proteins show the same features as CWPs except that they have a trans-membrane domain [20, 21].

Cell wall proteomics require challenging strategies comprising several steps, from the extraction to the identification of the proteins, compared to other sub-cellular proteomics works. Despite the technical hurdles, a lot of studies have been successful [8, 9]. Several aerial organs have been studied in different plant species, such as alfalfa [22], *Linum usitatissimum* [23], *Solanum tuberosum* [24], and *Arabidopsis thaliana* [25]. In *Brachypodium distachyon* leaves and stems, different classes of proteins have been identified and it was possible to address some of them to the mechanism of 2G biofuel production [26]. It is then possible to alter their expression to improve cell walls deconstruction, such as the upregulation of a cell wall transcript in rice [27].

In a recent publication, 69 CWPs have been described from isolated cells obtained from cell suspension cultures of sugarcane [28]. However, the description of the cell wall proteome from a differentiated organ is still missing. In this work, two different strategies were developed to extract the CWPs of two month-old stems: either a destructive method (DT Method) or a non-destructive one (ND Method), *i.e.* vacuum infiltration [29]. Proteins were identified by mass spectrometry (MS) and bioinformatics. The results were compared regarding the number and the type of CWPs. Quantitative MS data were used to identify the most abundant CWPs in sugarcane culms.

## Results

### Extraction of proteins from cell walls

Two-month-old sugarcane culms were selected for presenting a soft and young material, at an early stage of development. The use of young organs could lead to the identification of proteins involved in cell wall expansion, thus clarifying the mechanisms that the plant itself uses to allow growth.

Sugarcane features four stages of development: (i) germination and emergence, (ii) tillering phase, (iii) grand growth period and (iv) ripening phase, when sugar accumulates [30]. The tillering phase begins about 40 days after planting and can last up to 120 days, being the early stage of plant development [31, 32]. In this work, plants were collected 60 days after planting, halfway from the maximum tillering, measuring around 40–50 cm in height from the bottom to the upper leaf. This age was also chosen to allow distinguishing leaves and culms visually.

The DT Method was a destructive one relying on the grinding of the material and its centrifugation in solutions of increasing sucrose concentration. On the contrary, the ND Method was a non-destructive one, since it maintained the cell structures intact while performing the extraction of CWPs by vacuum infiltration of the tissues. Thus, it was expected that the DT Method would be able to extract more wall-bound proteins than the ND one. In both protocols, protein extraction from cell walls was performed using 0.2 M CaCl_2_ and 2 M LiCl. The efficiency of CaCl_2_ to release CWPs could rely on the fact that demethylesterified homogalacturonans strongly chelate calcium [33], solubilizing weakly-bound proteins by a competition mechanism [34]. On the other hand, LiCl was used to extract mostly hydroxyproline-rich glycoproteins [35] All the experiments were performed in duplicates.

**Fig. 1:**
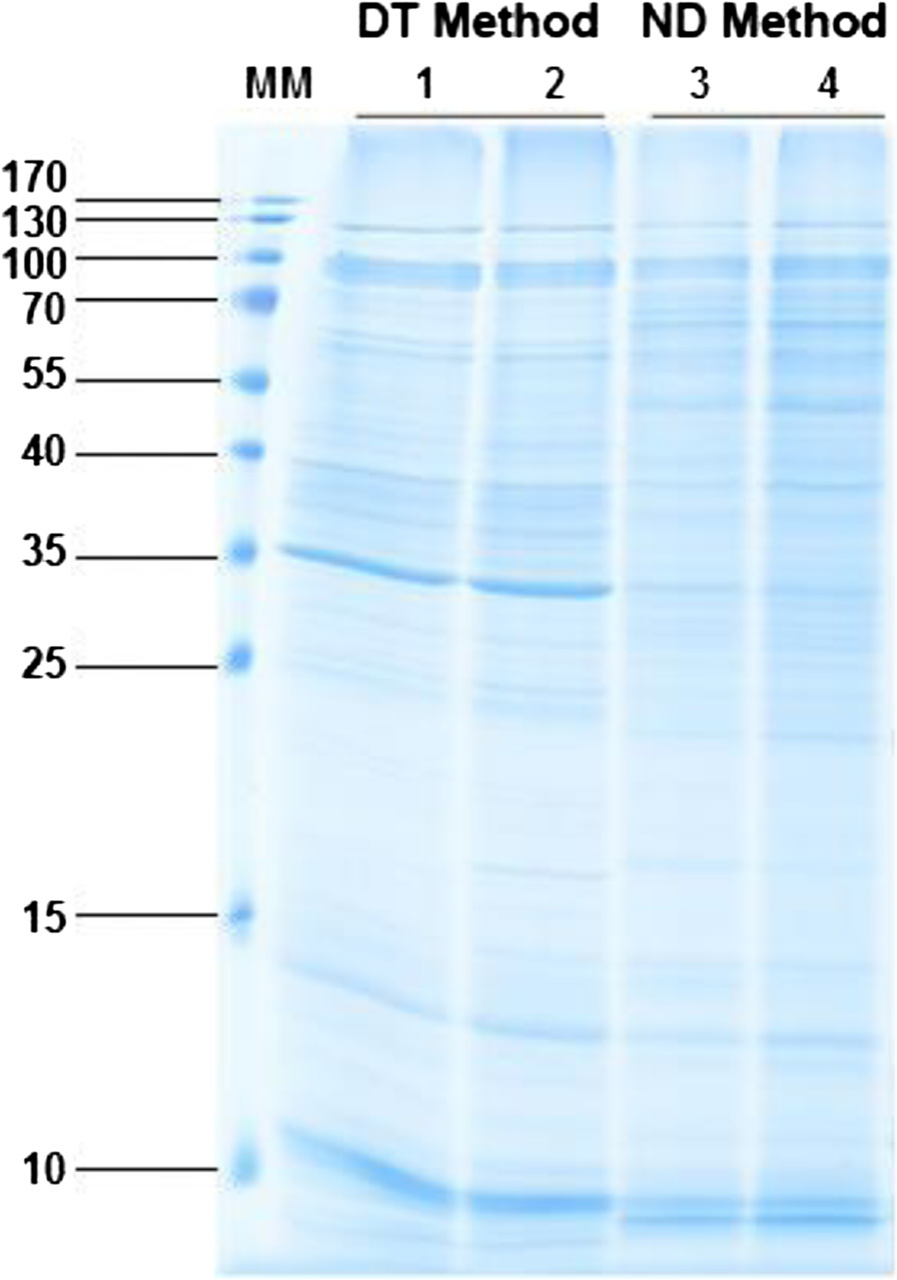
1D-electrophoresis of proteins extracted form 2-month-old sugarcane culm cell walls. Proteins have been extracted using either the DT or the ND Method. The biological repeats corresponding to each Methods are respectively numbered 1–2 and 3–4. The molecular mass markers (MM) are indicated in kDa on the left

The DT Method produced around 518 μg of proteins from 35 g of culms (fresh weight). Regarding the ND Method, the yield was slightly lower: around 667 μg of proteins were recovered from about 50 g of culms (fresh weight). Figure 1 shows the patterns of the proteins extracted from sugarcane culms. The presence of thin resolved bands after staining showed the quality of the procedure with no degradation pattern. Each biological replicate, using either method, showed a pattern very similar to that of its counterpart and each method gave rise to a different pattern.

### Identification of proteins by MS^E^ and bioinformatics analyses

Proteins were analyzed by shotgun LC-MS/MS, after tryptic digestion. The identification of proteins was performed using the translated-SUCEST database containing ESTs [36]. Homologous genes in *Sorghum bicolor*, the closest related species with a fully sequenced genome, were systematically searched for. Predictions of sub-cellular localization and functional domains were done on translated ESTs when they were full-length, otherwise on homologous *S. bicolor* coding sequences. Because of the high level of ploidy of the sugarcane genome [37], in some cases, different ESTs matched the same *S. bicolor* gene.

More detailed results of MS analyses, such as protein score and number of matched peptides, can be found in Additional files 1, 2, 3 and 4. About 65 % and 82 % of the proteins identified were found in both biological replicates, in the DT and ND Methods, respectively. These Methods allowed the identification of 70 and 103 different proteins from the translated-SUCEST database, respectively. From these, 39 (56 %) and 69 (67 %) proteins respectively had a predicted signal peptide, no known intracellular retention signal such as an endoplasmic retention signal and one trans-membrane domain at most (Table 1). These proteins were considered as CWPs (Additional file 5), and the others as intracellular proteins (Additional file 6). The DT and ND Methods lead to the identification of different sets of proteins.

**Table 1:** CWPs identified in sugarcane young culms

SUCEST accession number^a^	Number of peptides^b^	Number of unique peptides^b^	Protein score	Femtomole average	*S. bicolor* homologues	Functional annotation	Extraction method
Proteins acting on polysaccharides							
*SCCCCL3001B10.b*	16; 16	3; 6	4368.653; 1207.559	87.40605	Sb01g010840.1	GH1	ND
SCJFLR1017E03	7; 8	1; 1	627.7585; 389.633	4.65435	Sb01g010840.1	GH1	ND
SCEQLB1066E08	11; 5	9; 2	1258.872; 452.9845	6.72165	Sb01g010825.1	GH1	ND
SCEQHR1082B01	9; 7	8; 6	2256.574; 388.3896	28.182652	Sb02g028400.1	GH1	ND
SCEZLB1007A09	18; 12	11; 8	4982.336; 2259.83	41.55905	Sb01g008030.2	GH3	ND
SCEQLR1093F09*	12; 18–20; 14	5; 8–6; 5	523.6582; 1309.333 – 3688.09; 10810.11	17.23065 – 50.3928	Sb01g008040.3*	GH3	DT - ND2
SCCCCL4009F05	20; 14	16; 12	10955.92; 8452.891	156.84746	Sb06g030270.1	GH3	ND
SCQSAM1030G04	3; 2	3; 2	8506.176; 6709.646	71.61725	Sb06g030270.1	GH3	ND
*SCQSRT2031D12*	10; 1	7; 9	1169.301; 2716.698	34.5685	Sb03g045490.1	GH17	ND
SCVPRZ3029G05	3; 2	2; 1	853.3968; 815.915	15.9117	Sb03g040600.1	GH18	ND
*SCJLLB2076C12*	9; 8	4; 6	3324.314; 1244.686	40.19725	Sb06g021220.1	GH19	ND
*SCEZRZ3015E11*	8; 5	6; 5	3106.044; 2091.409	55.74035	Sb01g048140.1	GH19	ND
*SCCCCL5004G07*	8; 8	5; 6	7937.636; 2173.338	76.40835	Sb10g000660.1	GH28	ND
SCJFRT1007G04	4; 2	1; 1	4752.977; 2164.125	31.240002	Sb10g000660.1	GH28	ND
SCCCCL6004H07	9; 8	7; 7	633.8843; 542.2719	19.80175	Sb01g040750.1	GH35	ND
SCVPRZ3029F03	6; 4	4; 4	1035.417; 246.8027	8.5248	Sb03g029700.1	Acyl esterase (homologous to AtPMR5)	ND
SCSGLR1025E03	5; 2–5; 8	4; 2–4; 8	460.239; 887.9516- 1045.544; 226,6407	19.8042 – 13.1516	Sb02g042780.1	Pectin methylesterase (carbohydrate esterase family 8, CE8)	DT – ND
Oxido-reductases							
*SCCCRZ1002B03*	8; 1	3; 2	708.6577; 447.837	12.9346	Sb01g041770.1	Prx homologous to SbPrx20	DT
*SCCCRT1001G12*	9; 9–14; 9	5; 6–7; 5	2689.641; 2574.119 – 10308.06; 9888.154	65.36725 – 77.029495	Sb04g008590.1	Prx homologous to SbPrx71	DT - ND
SCCCLB1004B09*	9; 16	4; 4	690.8371; 1079.03	26.908451	Sb10g027490.1*	Prx homologous to SbPrx139	DT
SCEQRT2030A04*	7; 12	1; 3	306.9711; 658.3382	7.35725	Sb10g027490.1	Prx homologous to SbPrx139	DT
*SCCCLR1C03A09*	12; 11	8; 7	845.2605; 759.2087	46.113102	Sb09g004650.1*	Prx homologous to SbPrx115	DT
SCCCLR1C05G08*	11; 11	5; 8	1494.011; 1461.387	66.7759	Sb03g024460.1*	Prx homologous to SbPrx65	DT
SCRLAD1042E05	6; 4–5; 2	1; 2–1; 1	2528.694; 933.4598 – 873.3041; 1444.467	17.7972 – 10.54785	Sb09g002740.1*	Prx homologous to SbPrx108	DT – ND
SCVPRZ2035F03*	11; 8–9; 5	6; 6–4; 3	2417.947; 1151.436 – 1401.302; 1033.822	42.28915 – 17.264	Sb09g002740.1	Prx homologous to SbPrx108	DT - ND
SCVPLB1020D03	2; 8	2; 7	372.5895; 854.9581	23.364399	Sb03g046760.1	Prx homologous to SbPrx68	DT
SCEPRZ1011A06*	7; 11–12; 6	3; 5–4; 3	866.9835; 940.5853 – 5970.014; 1026.785	17.7399 – 45.46655	Sb03g010250.1*	Prx homologous to SbPrx54	DT - ND
**SCCCAD1001B08**	3; 3	1; 1	9547.608; 3981.619	Identified	Sb03g010740.1	Prx homologous to SbPrx55	ND
**SCJFRZ2013F04**	7; 1	1; 1	23916.97; 9892.667	7.78455			ND
**SCJLRT1019B02**	9; 6	1; 1	16559.67; 6310.499	14.36725			ND
*SCEQRT1024D03*	1; 1	3; 2	16709.58; 4972.862	60.093697	Sb03g010740.1	Prx homologous to SbPrx55	ND
*SCCCAD1001C08*	6; 5	3; 4	7855.956; 13571.95	28.358952	Sb02g042860.1	Prx homologous to SbPrx47	ND
SCQSST3114C09	5; 8	5; 4	2082.782; 583.5138	16.338501	Sb01g031740.1	Prx homologous to SbPrx14	ND
*SCBFFL4112F05*	2; 3	1; 2	1435.365; 4620.795	35.5025	Sb06g018350.1	Blue copper binding protein (plastocyanin)	DT
*SCRFHR1006G03*	3; 2	2; 1	391.4637; 1712.47	1.22305	Sb01g010510.1	Blue copper binding protein (plastocyanin)	DT
SCJLLR1104H07	3; 3	3; 3	912.4164; 417.4785	15.684	Sb07g011870.1	blue copper binding protein (plastocyanin)	ND
SCEPAM1021H07	8; 3	3; 3	924.3445; 347.9209	12.423151	Sb10g027270.1	Multicopper oxidase	ND
Proteins related to lipid metabolism							
*SCCCAM2002F12*	4; 5–4; 5	1; 1–1; 1	716.9828; 1069.318 – 5961.475; 9251.332	19.3443 – 115.4035	Sb03g038280.1	LTP	DT – ND
***SCBFLR1046E09***	5; 6–4; 5	1; 1–1; 1	816.9942; 1488.674 – 14868.01; 16290.19	34.07175 – 242.60735	Sb03g038280.1	LTP	DT - ND
***SCVPRZ2039B03***	5; 6–4; 5	1; 1–1; 1	816.9942; 2065.907 – 14868.01; 16290.19	Identified - identified			DT - ND
***SCVPRZ2041C11***	5; 6–4; 5	1; 1–1; 1	902.0069; 1488.674 – 18617.13; 24758.37	8.77335 - identified			DT - ND
***SCCCLR1072C06***	3; 2–6; 5	1; 1–1; 1	243.1648; 2495.928 – 35001.54; 23317.92	6.5461 – 132.9761	Sb08g002700.1	LTP	DT - ND
***SCRFLR1012A10***	3; 2–7; 5	1; 1–1; 1	345.6155; 2314.583 – 35158.61; 23317.92	Identified - identified			DT – ND
***SCEPRT2047G01***	10; 5	1; 1	35204.81; 23919.76	250.87096			ND
***SCEZLR1031G07***	5; 4	1; 1	34256.59; 23296.33	Identified			ND
***SCRUSB1064D08***	9; 5	1; 1	35280.34; 23317.92	Identified			ND
*SCEPLB1044H04*	3; 4–3; 4	1; 1–1; 1	3924.14; 3159.724 – 1548.66; 926.7365	189.36455 – 46.1548	Sb01g049830.1	LTP	DT – ND
*SCEZLB1006F09*	3; 4–3; 2	1; 1–1; 1	6772.019; 11919.76 – 8845.361; 13343.73	194.30121 – 146.9349	Sb08g002670.1	Protease inhibitor/seed storage/LTP family	DT - ND
SCCCLR1048F06 - **SCCCLR1048F06**	10; 13–5; 4	1; 1–1; 2	91432.66; 77846.23 – 176534.4; 124470.6	318.6974 – 352.27365	Sb08g002690.1	Protease inhibitor/seed storage/LTP family	DT - ND
***SCBGLR1114E07***	5; 5	2; 2	145690.4; 125905.7	588.4425			ND
**SCCCCL3004H07.b**	3; 3	2; 2	145392.5; 124459	Identified			ND
***SCVPHR1092G06***	4; 4	2; 2	145392.5; 124470.6	Identified			ND
*SCUTST3131G03*	3; 6–4; 3	2; 1–1; 1	6793.345; 3745.457 – 21630.89; 20854.45	150.85635 – 109.76019	Sb08g002690.1	Protease inhibitor/seed storage/LTP family	DT – ND
SCCCCL3001E03.b*	5; 7	2; 3	3263.44; 1945.298	39.72605	Sb01g033830.1*	LTP	ND
***SCJFRZ2033G07***	4; 3	1; 1	26826.32; 13939.5	8.15625	Sb08g002700.1	LTP	ND
***SCRUFL4024B04***	4; 3	1; 1	26805.06; 13987.92	202.91615			ND
SCCCRZ1001H02	3; 3	1; 1	7741.618; 3045.282	70.59645	Sb03g039880.1	LTP	ND
***SCCCRZ2002G09***	5; 5	2; 2	20532.6; 6676.209	49.378	Sb06g016170.1	LTP	ND
**SCQSFL3039E08.b**	5; 5	2; 2	22572.43; 7334.285	20.2533			ND
SCCCLR1024C05*	6; 3	1; 1	11552.57; 5130.042	5.59605	Sb08g002660.1*	Protease inhibitor/seed storage/LTP family	ND
***SCCCLR1076D05***	6; 5	1; 1	16880.65; 8523.134	129.08115			ND
SCEPLB1044H11*	7; 3	1; 1	11788.22; 5527.095	13.30225			ND
*SCCCLR2C03F01*	3; 3	1; 1	9426.873; 6414.531	78.86415	Sb08g002670.1	Protease inhibitor/seed storage/LTP family	ND
SCCCRT1003B03	6; 4	2; 3	722.451; 408.6039	26.09375	Sb10g003930.1	GDSL lipase	ND
Proteases							
SCBGLR1023G11	6; 8	5; 8	553.142; 705.4267	24.15155	Sb04g029670.1	Asp protease, peptidase A1	DT
SCBGLR1097G03	4; 6	3; 3	1475.072; 7045.55	168.19795	Sb05g027510.1	Asp protease, peptidase A1	DT
SCMCLR1123H12	6; 7–3; 2	3; 3–2; 1	1722.723; 4799.767 – 598.7939; 1717.224	122.60135 – 52.145752	Sb05g027510.1	Asp protease, peptidase A1	DT - ND
SCQGST1032H01	11; 14	8; 7	653.9818; 997.1608	45.5457	Sb05g027510.1	Asp protease, peptidase A1	DT
SCQGSB1083B11	8; 5	5; 4	4851.335; 4946.649	47.901802	Sb02g041760.1	Asp protease, peptidase A1	ND
SCRLRZ3042B09	9; 6	5; 3	390.5894; 367.0505	7.24305	Sb03g026970.1	Asp protease, peptidase A1	ND
SCVPLR2012E01	3; 3–4; 3	2; 2–2; 2	1075.957; 4197.95 – 23533.5; 11935.54	147.1347 – 160.93646	Sb01g044790.1	Asp protease/Taxi _N/Taxi_C	DT - ND
SCVPRZ2038B09	3; 4–4; 2	2; 3–4; 2	1194.285; 2283.918 – 6719.425; 1320.476	61.6057 – 103.41875	Sb01g044790.1	Asp protease/Taxi _N/Taxi_C	DT - ND
*SCCCST1004B07*	11; 8	11; 8	4096.934; 3149.51	44.2912	Sb01g013970.1	Ser protease (subtilisin family, peptidase S8/S53)	ND
SCJFRZ2011B07	5; 4	4; 3	2390.547; 1211.642	25.90875	Sb06g016860.1	Ser protease (subtilisin family, peptidase S8/S53)	ND
SCCCLR1022B11*	7; 5	6; 6	1017.185; 492.4018	20.1544	Sb06g030800.1*	Cys protease, (papain family, peptidase C1A)	ND
Proteins with interaction domains (with proteins or polysaccharides)							
**SCJFLR1013A04**	4; 4	1; 1	3741.598; 4709.589	31.54085	Sb05g026650.1	Ser protease inhibitor (Bowman-Birk)	DT
**SCRUFL3062D08 -** SCRUFL3062D08	5; 5–4; 4	1; 1–1; 1	2784.365; 4868.514 – 11731.47; 8790.486	45.9138 – 44.6385			DT - ND
Signaling							
SCRUAD1063C06	4; 2	4; 4	5824.59; 1313.647	55.09195	Sb09g000430.1	Leucine-rich repeat (LRR) receptor kinase	ND
Miscellaneous proteins							
SCEZRZ1014C04*	6; 5–4; 8	2; 2–1; 1	6025.488; 9344.188 – 18335.89; 4427.775	79.66205 – 67.5792	Sb03g039330.1*	Thaumatin	DT - ND
***SCCCLR2003G06***	4; 4	1; 2	1364.424; 533.9335	18.352499	Sb08g018720.1	Thaumatin	ND
**SCUTLR1037F02**	3; 4	1; 2	1083.055; 546.6533	Identified			ND
*SCCCSD1003E02*	3; 2	1; 1	2326.563; 4054.495	18.28735	Sb08g022410.1	Thaumatin	ND
*SCRUHR1076B06*	3; 2	1; 1	3641.288; 5434.818	2.588	Sb08g022410.1	Thaumatin	ND
*SCVPRT2073B04*	4; 4	2; 2	2289.087; 18490.86	87.17195	Sb08g022420.1	Thaumatin	ND
*SCBGRT1047G10*	6; 7	4; 6	3131.626; 2684.819	52.720253	Sb02g004500.1	Germin (cupin domain)	ND
*SCCCLR2C02D04*	3; 4	3; 3	9021.729; 14936.96	149.43965	Sb09g004970.1	Germin (cupin domain)	ND
SCCCRZ1C01H06	13; 1–12; 14	4; 3–7; 6	3376.122; 3105.723 – 10082.07; 3506.265	55.5037 – 33.9362	Sb08g001950.1	Nucleoside phosphatase	DT - ND
SCJLRT3078H06	6; 2	3; 1	1286.289; 1236.944	45.866447	Sb05g025670.1	Dirigent protein	DT
SCVPRT2073B08	6; 4	4; 1	333.12; 1046.494	19.9186	Sb10g001940.1	SCP-like extracellular protein	ND
Unknown function							
SCCCCL4009G04*	11; 1–8; 8	4; 4–3; 3	1383.823; 3928.575 – 15270.52; 16670.59	126.7531 – 141.14679	Sb01g004270.1*	Unknown function (DUF642)	DT - ND
SCSGLR1084A12*	12; 14–10; 9	6; 6–6; 5	6772.538; 5277.542 – 18024.07; 13067.63	158.43881 – 150.2402	Sb01g004270.1	Unknown function (DUF642)	DT - ND
SCCCLB1001G04	7; 3	5; 3	314.0911; 273.5209	9.64495	Sb03g027650.1	Unknown function (DUF642)	DT
SCVPLR2027A11	5; 4–5; 3	5; 4–2; 1	2870.491; 1609.635	34.981	Sb07g026630.1	Unknown function (DUF568)	ND
SCCCRZ3002G10*	4; 7–6; 5	1; 1–1; 2	844.0264; 313.8415 – 4025.449; 1307.088	3.07575 – 35.7293	Sb01g031470.1*	Homologous to phloem filament protein 1 (*Cucurbita* phloem)	DT - ND
SCEZRT2018F03*	4; 6	1; 1	675.4673; 446.4631	5.89995	Sb01g031470.1	Homologous to phloem filament protein 1 (*Cucurbita* phloem)	DT
SCEZLB1013B06	14; 15	8; 11	5254.302; 4812.086	137.18835	Sb10g001440.1	Homologous to phloem filament protein 1 (*Cucurbita* phloem)	DT
*SCSFST1066G10*	5; 5–6; 5	1; 1–1; 1	718.6985; 2383.583 – 15310.67; 5735.458	102.53975 – 192.7034	Sb08g018710.1	Expressed protein	DT - ND
SCRUFL4024B08.b	3; 6	1; 2	5551.887; 12727.94 – 52010.09; 28346.53	120.5459 – 288.07706	Sb08g018710.1	Expressed protein	DT - ND
SCCCRZ2004B02*	8; 8	1; 1	9551.813; 4751.091	43.9473	Sb03g000700.1*	Expressed protein	ND
*SCCCLR1079C11*	7; 4	6; 4	3527.088; 4063.023	33.617348	Sb04g011100.1	Expressed protein	ND
**SCAGLR2011E04/SCEPAM2057B02**	3; 3	1; 1	11178.66/11961; 8954.521/7215.72	identified	Sb08g003040.1	Expressed protein (stress responsive alpha/beta barrel)	ND
**SCEPLR1051E09**	3; 3	1; 1	11178.66; 7215.72	28.171349			ND

^a^Bold letters indicate that the ESTs share common sequences. Full length ESTs are in italics. Stars (*) indicate the proteins also identified in the cell wall proteome of sugarcane cell suspension cultures [15]

^b^Semicolons separate data from different biological repeats. Dashes separate data from different extraction methods (DT, then ND)

**Fig. 2:**
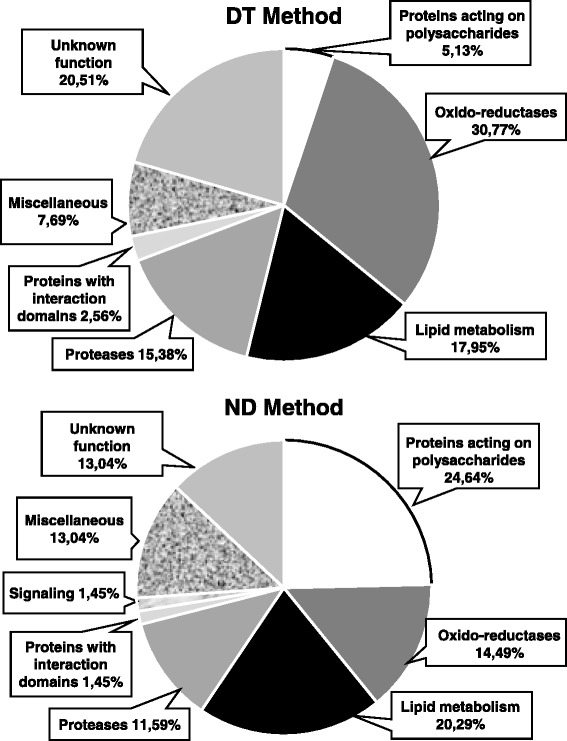
Distribution of CWPs identified in 2-month-old sugarcane culms. Proteins were distributed in functional classes according to bioinformatics predictions: PAC stands for proteins acting on carbohydrates; OR, for oxido-reductases; LM, for proteins possibly involved in lipid metabolism; P, for proteases; ID, for proteins with interaction domains (with proteins or polysaccharides); S, for proteins possibly involved in signaling; M, for miscellaneous; UF, for unknown function

Altogether, 84 different CWPs were identified and distributed into eight functional classes (Fig. 2 and Table 1): proteins acting on carbohydrates, proteins possibly related to lipid metabolism; proteins with interaction domains; oxido-reductases; proteases; miscellaneous proteins; signaling and proteins of unknown function. From these 84 CWPs, 24 (29 %) were identified using both the DT and ND Methods. It should be noted that no structural protein was identified. Besides, 16 CWPs (18 %) were previously identified in the cell wall proteome of sugarcane cell suspension cultures [28]. Consequently, 68 sugarcane CWPs were newly identified in this study.

Regarding the DT Method, the oxido-reductases (31 %), mainly peroxidases (Prxs) and two blue copper binding proteins, constituted the most represented class, followed by proteins related to lipid metabolism (18 %), all being lipid transfer proteins (LTPs). Asp proteases (16 %) and miscellaneous proteins (7.5 %), comprising thaumatin, germins and dirigent protein, were also identified (Table 1). Surprisingly, only one glycoside hydrolase (GH) of the GH3 family, as well as a single pectin methylesterase (PME) were identified from the proteins acting on carbohydrates class (5 %). Proteins with interaction domains (2.5 %) were represented by one serine protease inhibitor. Proteins of yet unknown function (20 %) were numerous and it was possible to highlight the presence of proteins with DUF642 domains, already found in other cell wall proteomes [38, 39], and proteins homologous to phloem filament protein 1.

The most represented functional class using the ND Method was that of proteins acting on carbohydrates (25 %), mostly GHs (families 1, 3, 19, 28, 17, 18, 35) and two carbohydrate esterases. Proteins related to lipid metabolism (20 %) comprised LTPs and one GDSL-lipase. Oxido-reductases (14 %) were mostly Prxs. Miscellaneous proteins (13 %) were mainly represented by thaumatins and germins. Proteases (12 %) were Asp, Ser or Cys proteases. Proteins with interaction domains were represented by one Ser protease inhibitor and signaling proteins by one leucine-rich repeat receptor kinase. Finally, proteins of unknown function comprised proteins with DUF642 and DUF568 domains.

We have also performed a quantitative analysis of the CWPs identified by both methods (Table 1). Only the proteins present in amounts higher than 100 femtomoles, calculated by averaging the results of the two biological repeats, have been listed in Table 2. When a protein has been identified using both methods, its quantification could be the same or different if either of the two methods could extract it more efficiently. These differences could, (*i*) result from the loss of proteins during the washings steps required to purify cell walls using the DT Method or, (*ii*) due to different types of interactions with cell wall components. Among the proteins present in high amount in culm cell walls, LTPs are well represented with 10 out of 17 proteins. One GH3, three Asp proteases and two DUF642 proteins were also found in the top17 list.

**Table 2:** Most abundant CWPs in the cell wall proteome of sugarcane young stems. Proteins with average amounts between the two biological repeats higher than 100 femtomols using either method are listed (see Table 1)

SUCEST accession number	Functional annotation	Method^a^
Proteins acting on carbohydrates		
SCCCCL4009F05	GH3	ND
Proteins related to lipid metabolism		
SCCCAM2002F12	LTP	DT < < ND
SCBFLR1046E09	LTP	DT < < ND
SCEPLB1044H04	LTP	DT > > ND
SCEZLB1006F09	LTP	DT > ND
SCCCLR1048F06	LTP	DT ~ ND
SCUTST3131G03	LTP	DT > ND
SCRUFL4024B04	LTP	ND
SCCCLR1076D05	LTP	ND
Proteases		
SCBGLR1097G03	Asp protease	DT
SCMCLR1123H12	Asp protease	DT > > ND
SCVPLR2012E01	Asp protease	DT ~ ND
Unknown function		
SCCCCL4009G04	Expressed protein (DUF642)	DT ~ ND
SCSGLR1084A12	Expressed protein (DUF642)	DT ~ ND
SCEZLB1013B06	Homologous to phloem filament protein 1	DT
SCSFST1066G10	Expressed protein	DT < < ND
SCRUFL4024B08.b	Expressed protein	DT < < ND

^a^The relative amount of proteins quantified using either method is indicated (see Table 1)

**Fig. 3:**
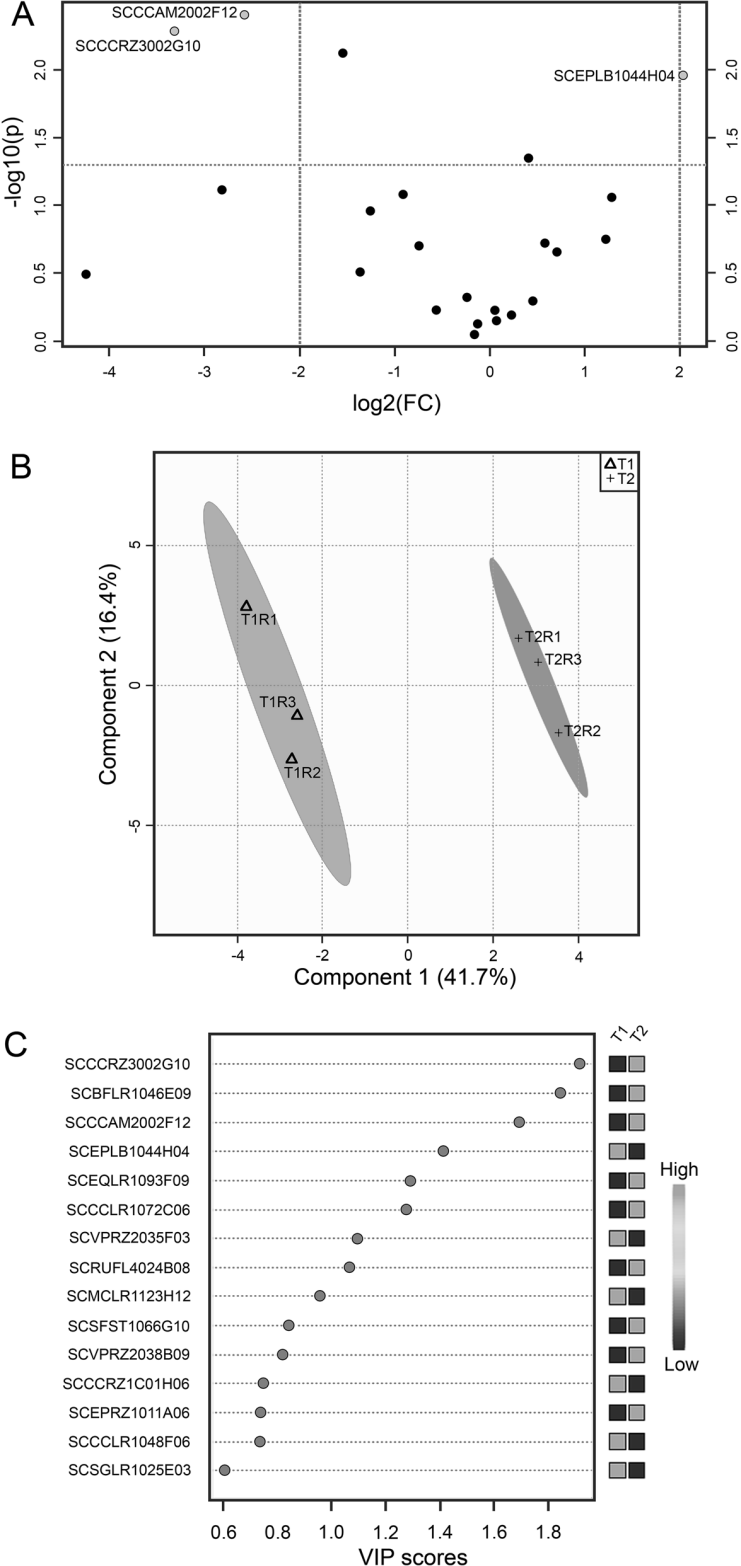
**a**. Volcano plot: Univariate Statistical analysis of the quantified proteins in both methods. Axis *x*: Fold Change. Axis *y*: *p* value. **b**. Scores plot: separation of two groups based on the statistical analysis of the amount of the proteins. **c**. VIP scores. Multivariate Statistical analysis showing the 15 proteins that contributed the most to the separation of the two groups: DT (T1) and ND Method (T2), regarding quantitative data. *Black squares* mean higher amounts of proteins and gray ones lower amounts. Since two replicates were used for each treatment, the median was calculated from both of them and named T1R3 and T2R3 for DT and ND Methods, respectively

Two approaches were used to statistical analysis: a multivariate analysis, the Scores plot and Vip scores (Fig. 3b, c, respectively), and a univariate one, the Volcano plot, as shown in Fig. 3a. In Fig. 3a, three proteins could be considered as those contributing the most to the distinction between the DT and ND Methods. Figure 3b indicates that the DT and ND Methods differ statistically from each other, since it is possible to separate two distinct groups of proteins regarding the quantity of proteins extracted in each technique. In addition, the two first components (vectors) contributed positively to the model (value of Q^2^ positive = 66.5 %), and the variation of the proteins was 97.5 % (R^2^). Values of Q^2^ > 0.08 indicates that a model is better than chance, and scores of 0.7 or higher, demonstrate a very robust trend or separation [40]. The protein SCCCRZ3002G10 of unknown function was the one that contributed the most to the separation of the groups, being found in higher amount using the ND Method (Fig. 3a, c). The SCCCAM2002F12 and SCEPLB1044H04 LTPs, in turn, were the third and the fourth proteins that contributed to the separation of the two groups in Partial-Least Squares Discriminant Analysis - PLS-DA2, being found in higher amount in the ND and DT Methods, respectively.

As presented in Fig. 3c, using the average of the quantitative data obtained for each method, the statistical analysis showed that from the 15 proteins that most contributed to distinguish the DT and ND Methods, nine of them showed a much higher amount using the ND Method. Additional file 7 shows important features identified by Volcano Plot.

### Comparison of the CWPs of sugarcane young culms to those of stems of other plants

**Fig. 4:**
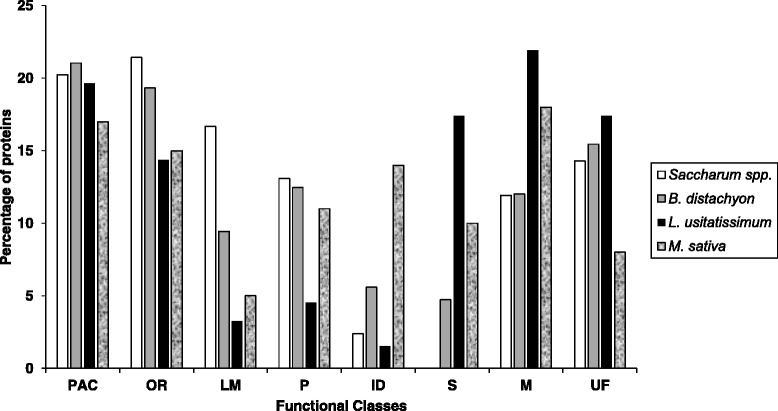
Comparison of the percentage of proteins identified. CWPs present in this study were compared with known cell wall proteomes of stems from different species: *B. distachyon* [26], *L. usitatissimum* [23], and *M. sativa* [22]. Proteins were distributed in functional classes – according to the legend of Fig. 2

Previous cell wall proteomics studies were performed on *B. distachyon* basal and apical internodes [26], *Medicago sativa* basal and apical stems [22] and *Linum usitatissimum* young stems [23]. All these data have been collected in the *WallProtDB* database [39] and annotated in the same way, thus allowing comparisons [41]. These CWPs were compared to the newly identified CWPs of sugarcane stems (Fig. 4). In *B. distachyon*, a protocol very similar to the DT Method was used, but the LC-MS/MS analysis were done with 1-D gel pieces [26]. *L. usitatissimum* stem CWPs were extracted using a protocol similar to the DT Method and 1-D gel pieces corresponding to stained protein bands were used as starting material for FT-ICR MS analysis [23]. On the other hand, in alfalfa stems, EGTA tretament and LiCl were used for protein extraction, and 1-D gel pieces were digested prior to analysis using a nanoAcquity UPLC system [22]. Although different strategies for protein extraction and MS analyses have been used, all the protocols used the same salts to extract proteins from cell walls: CaCl_2_ and/or LiCl.

The stem cell wall proteomes of all the above species showed very similar percentages of proteins acting on carbohydrates. An outstanding observation was that sugarcane had a much higher percentage of proteins related to lipid metabolism (17 %) than all the other species (0–9 %). The dicot *M. sativa* presented a much higher proportion of proteins with interaction domains in comparison with the monocots (14 % *vs* less than 5 %). The monocots showed a higher proportion of oxido-reductases in comparison with the dicots (about 20 % *vs* about 15 %). A much smaller proportion of proteases was found in *L. usitatissimum* stems [23].

## Discussion

In this work, 84 different sugarcane CWPs were identified in young culms using two different strategies. Together with the cell wall proteome of cell suspension cultures [28], 137 different CWPs of sugarcane have been identified. In this study alone, 68 CWPs were newly identified and 16 CWPs were identified in both culms and cell suspension cultures, among which 5 Prxs. Besides, the proportion of proteins predicted to be intracellular in culm extracts (33 % and 44 %) was lower than in sugarcane cell suspension culture extracts (81.6 %) [28], being quite the same as in *B. distachyon* young internodes [26]. This is probably inherent to the type of material, since a lot of cell debris are present in the culture medium [28].

Interestingly, the proportion of intracellular proteins was higher in leaves than in stems in *B. distachyon* [26]; the same case has been observed for sugarcane (unpublished observations). The ND Method has lead to the identification of about 75 % more CWPs than the DT Method (69 CWPs *vs* 39), and around 81 % of the CWPs (68 CWPs out of 84) have been identified using one method of extraction only. These results show the importance of using different strategies to enlarge the coverage of a cell wall proteome. The ND Method has allowed the recovery of more CWPs of sugarcane culms, and much more GHs than the DT method. If the objective of the study is to get an overview of CWPs or of glycosidases, this strategy should be considered. In addition, if the goal is especifically to recover GHs, perhaps a total protein extraction followed by affinity chromatography on Concanavalin A is the best option [25]. However, if the aim is to go deeper into Prxs, the DT Method looks more appropriate. Besides, both methods showed a good reproducibility since between 65 % and 82 % of CWPs were identified in both biological replicates. Although rarely discussed in cell wall proteomics paper, this result is consistent with those of previous studies [26].

The ND Method could recover both a higher number of CWPs and a higher amount of those contributing to the discrimination between the two methods through the statistical analysis. Additionally, the three proteins highlighted in the univariate analysis were also present in the multivariate analysis, being numbers 1, 3 and 4 from the 15 CWPs considered to be the most important for the discrimination between the two methods. The major difference between the two ND and DT methods regards proteins acting on carbohydrates: only one CWP has been identified using the DT Method whereas one fourth of the CWPs belongs to this class using the ND Method. Since the same organs were analyzed, this difference has to be related to the strategy used for protein extraction. Some proteins could have been lost during the washing steps required to clean cell wall fragments in the case of the DT Method [9]. This could explain why more CWPs were found using the ND Method. However, the use of the DT Method with sugarcane cell suspension cultures allowed the recovery of several GHs [28]. Then, the low number of GH identified in this study using the DT Method could be related to the structure of the sugarcane culm cell walls. In the case of grasses, cell walls contain different matrix polysaccharides and protein components, when compared to dicot cell walls. As an example, grass cell walls present cellulose microfibrils interlocked by glucuronoarabinoxylans instead of xyloglucans. In addition, they contain a substantial proportion of non-cellulosic polymers wired on cellulose microfibrils by alkali-resistant phenolic linkages [7].

As found with the ND Method, most previous cell wall proteomics studies showed that proteins acting on carbohydrates were the most represented [29, 42]. The role of such proteins in cell walls points to the rearrangements of polysaccharides during development [11, 43–45]. These modifications can occur through the hydrolysis of glycosidic bonds within polysaccharides or between a carbohydrate and a non-carbohydrate moiety [46]. Not surprisingly, they can play important roles during germination [47], defense against herbivory [48], lignification [49] and regulation of phytohormones [50]. In this functional class, all but two were GHs, represented by one acyl-esterase and one PME. GH1, 3, 17, 19 and 28 were also found as the major GH families present in the cell wall proteomes of *B. distachyon*, *Oryza sativa* and *A. thaliana* [26, 51]. One member of the *A. thaliana* GH1 family has been shown to degrade β-mannosides, suggesting that it could hydrolyze mannans, galactomannans, or glucogalactomannans *in muro* [46]. Proteins of the GH3 family could have *α*-L-arabinofuranosidase and/or *β*-xylosidase activities [52]. One GH3 is among the most abundant CWPs identified in sugarcane culms. GH1 and GH3 were also identified in termite stomach, being characterized as β-glucosidases, *i.e.* cellulases that preferentially hydrolyze β-1-4 glycosidic linkages [53]. However, the overexpression of a rice β-glucosidase and an endo-glucanase was null and led to deleterious effects, respectively [54]. This may indicate that perhaps these enzymes should not be altered if the goal is to achieve a less recalcitrant plant. However, by altering the expression of exo-glucanases, it was possible to increase saccharification in rice, besides negative effects on plant development [54]. In *B. distachyon* culms, no GH35 was identified and a higher proportion of GH17 and 18 were found in comparison to GH1 and 3 [26], an opposite finding to sugarcane culms. Another CWP-to-watch is the PME, since the expression of a fungal pectin methylesterase inhibitor (PMEI) in wheat and *Arabidopsis* could increase the efficiency of enzymatic saccharification [55].

The proportion of oxido-reductases was almost the same as that found in the cell wall proteome of sugarcane cell suspension cultures [28]. In *B. distachyon* culms, the percentage of oxido-reductases was closer to that found using the ND Method [26], although the work was performed with a protocol very similar to the DT Method. So it is not possible to conclude that the method itself was more likely to extract these proteins. As found in *B. distachyon* [26], Prxs and blue copper binding proteins were more numerous in the sugarcane than in the *A. thaliana* cell wall proteome. Different populations of Prxs were extracted by the ND and DT Methods. This could be related to their different abilities to interact with pectins as shown for a zucchini and an *A. thaliana* Prxs [56, 57]. Although Prxs are numerous (14 out of 84 CWPs) in the sugarcane culm cell wall proteome, none of them was found amongst the most abundant CWPs. Prxs are well-known cell wall enzymes, identified in many cell wall proteomics studies [58]. They could be involved in cell wall polysaccharide rearrangements during development, defense reactions or signaling [58]. Their activity is versatile. During the hydroxylic cycle, Prxs can produce ROS that break cell wall polysaccharides in a non-enzymatic way, promoting wall extension, whereas during the peroxidative cycle, Prxs can favor cross-linking of cell wall components such as structural proteins or lignins [59]. So, Prxs are also a class of proteins to be watched when searching for proteins that could potentially facilitate the production of cellulosic ethanol. The blue copper binding proteins have already been found in cell wall proteomes [42, 60]. They have been associated to redox processes such as electron transfer proteins with small molecular mass compounds [61]. Blue copper binding proteins were not found in the cell wall proteome of sugarcane cell suspension culture [28].

LTPs were already identified in many cell wall proteomes [29, 60]. They have been assumed to bind hydrophobic molecules in cell walls which could be essential for cell wall loosening, thus facilitating wall extension [62]. LTPs could also be involved in cuticle formation [63]. Since sugarcane culms have a thick cuticle, this could explain the high number of identified LTPs in their cell wall proteome. This number is much higher than in any other species studied before [10, 22, 25, 26]. This explanation is consistent with the fact that a much lower number of lipid-related proteins was found in sugarcane cell suspension cultures which are undifferentiated cells [28]. LTPs are also the family that embraced the highest number of proteins with an average quantity higher than 100 femtomoles (8 out of 17 proteins). Additionally, five LTPs were among the 15 proteins who contributed the most to the discrimination between the ND and DT Methods.

Proteases can participate in various processes of the plant life cycle, such as development, defense, stress response and adaptation to the environment [64]. In sugarcane culms, mostly Asp proteases have been identified. Asp proteases were also numerous in the *B. distachyon* cell wall proteome [26]. Three Asp protease were found among the 17 most abundant CWPs of the sugarcane culm cell wall proteome. Asp proteases may be linked to disease resistance signaling, being accumulated in the extracellular matrix under pathogen attack [65]. Besides, two Ser proteases of the subtilisin family were identified. Proteins of this family have been shown to display various functions in plant development and signaling [14, 64, 66, 67]. Finally, one Cys protease was identified, a type of protease that can be related to the regulation of senescence and seed germination, as well as to defense roles [65, 68]. Cys proteases are known to be secreted in the apoplast [65]. It should be noted that Ser and Cys proteases were only found using the ND Method.

Several thaumatins have been identified, mainly using the ND Method. Thaumatins are pathogenesis-related proteins. Several of them have been shown to be β-1,3-glucanases showing anti-fungal activity [69]. However, one thaumatin has been shown to exhibit a polyphenol oxidase activity [70]. Finally, some proteins of unknown function were found, especially members of the DUF642 and DUF568 families. DUF642 proteins present a conserved region found in a number of plant proteins [71], and have been identified in all the cell wall proteomes studied so far [63]. One *A. thaliana* DUF642 protein has been shown to interact with cellulose *in vitro* [38]. In sugarcane culms, two DUF642 proteins were among the most abundant proteins. Thus, these proteins probably take part in important processes in the cell wall. On the other hand, one DUF568 is known as an auxin-responsive protein, AIR12, that may interact with other redox partners within the plasma membrane to constitute a redox link between the cytoplasm and the apoplast [72].

Some protein families were under-represented in the cell wall proteome of sugarcane culms when compared to other cell wall proteomes. Only one protease inhibitor has been identified. It belonged to the Bowman-Birk family. It has been characterized as a trypsin inhibitor associated with the regulation of endogenous seed proteinases, storage of sulfur amino acids and defense against insects and pathogens [73]. In sugarcane cell suspension cultures, different families of proteins with interaction domains have been identified, and in *B.distachyon*, proteins of the Bowman-Birk family were found both in leaves and internodes [26]. Regarding proteins possibly involved in signaling, the LRR receptor kinase family was commonly found in other cell wall proteomics studies [23, 26, 28]. Such proteins probably play roles in signal perception during development or in response to environmental cues [74]. One dirigent protein has been identified in sugarcane culms. Such proteins have been assumed to play a role in lignification through the control of monolignol coupling affecting wall flexibility and its mechanical strength [75]. Members of this family have been identified in *B. distachyon* stems [26]. No structural protein has been found in the sugarcane cell wall proteome, as in previous studies [23, 25, 26, 28, 29, 42]. This is probably because these proteins are difficult to extract when they are covalently cross-linked [59]. Usually, they cannot be extracted by salts [35], thus, different strategies should be used if structural proteins, such as extensins, are the focus [76].

## Conclusions

This work has contributed to three main aspects: (*i*) characterize CWPs from sugarcane young stems, (*ii*) compare the CWPs found, regarding type and amount, using two different methods of extraction and (*iii*) point at candidate CWPs to be used in future research to enhance 2G ethanol production. This study also offered a glimpse to the quantification of CWPs, providing help for the decision of which method is more suitable for the efficient extraction of different types of CWPs from sugarcane culms. If the focus is on GHs or getting an overview of the cell wall proteome, then the ND Method could be used. Otherwise, if looking for Prxs, the DT Method is the more adequate. Our results highlight the importance of using different strategies to isolate CWPs.

Future studies that could explain how these proteins interact with cell wall components, and use these GHs to obtain a custom-made plant to enhance 2G ethanol production will bring new perspectives to an old problem: the viability of this biofuel. In addition to GHs, attention should be paid to other proteins such as Prxs and dirigent proteins, since Prxs can favor cross-linking of the cell wall components such as proteins or lignins [58]. Therefore, they could be used in genetic engineering since lignin is a cell wall barrier preventing the access of cellulose to enzyme attack in order to break these sugars into fermentable ones [77]. Lowering the lignin content or modifying lignin linkages to facilitate its removal are two possible ways to enhance the efficiency of biomass deconstruction [1]. Finally, some proteins of yet unknown function could be interesting candidates.

## Methods

### Plant material

Sugarcane plants from variety SP80-3280 were used in all the experiments, provided by Dr. Maria Cristina Falco from the Sugarcane Technology Center (CTC, http://www.ctcanavieira.com.br/). This sugarcane variety was chosen as the one having available sequenced ESTs [36]. Pieces of culms of 7 cm each containing lateral buds were planted in pots, containing a mixture of vermiculite 1:1 compost (Plantmax, Eucatex Indústria e Comércio SA, São Paulo, Brazil) and acclimated in a greenhouse at 26 °C. Sugarcane plants were watered daily and nutrient solution (Plant-Prod 4 g/L, Master Plant-Prod Inc, Brampton, ON, Canada) was added every 15 days. Since the plants were obtained after only two months of growth, all the portions of the culms were collected. For both methods, the plants were collected and the proteins were immediately extracted.

### Extraction of proteins from cell walls and separation by 1D-electrophoresis

Two different strategies were used, respectively called DT and ND Method. Two biological replicates were performed in each case. For each experiment, material from 2 different plants randomly picked was used. The DT Method was a destructive one [35], whereas the ND Method was a non-destructive one [29].

To perform the extraction of proteins with the DT Method, culms were collected and cut into small pieces, washed with Ultra High Quality (UHQ) water and transferred to a blender containing 500 mL of a sodium acetate buffer 5 mM, pH 4.6, with 0.4 M sucrose, polyvinylpolypyrrolidone (PVPP) (1 g per 10 g of fresh tissue, Sigma Chemical, St Louis, MO, USA) and 3.3 % (v/v) anti-protease cocktail (P9599, Sigma). The plant material was ground in the blender for 8 min at maximum speed. Cell walls were separated from the soluble cytoplasmic fluid through centrifugation for 15 min, at 1000 *g* and 4 °C. The resulting pellet was submitted to two successive centrifugations in 500 mL of sodium acetate buffer 5 mM, pH 4.6, plus 0.6 M and 1 M sucrose, respectively. The final pellet was washed with 3 L of 5 mM sodium acetate buffer, pH 4.6, on a Nylon net (pore size = 50 μm) (Nitex, Dominique Dutscher, Brumath, France). The resulting cell wall fraction was ground with liquid nitrogen, and freeze dried for 48 h. The extraction of proteins from purified cell walls with 0.2 M CaCl_2_ and 2 M LiCl solutions was conducted as described [59].

In the case of the ND Method, the culms were collected, washed with UHQ water, cut in small pieces (about 7 cm in length) and then immersed in a beaker with a buffer solution containing 5 mM sodium acetate, pH 4.6, 0.3 M mannitol, 0.2 M CaCl_2_ and 0.1 % (v/v) anti-protease cocktail (P9599, Sigma). The beaker was placed in a desiccator attached to a vacuum pump and the culm pieces were infiltrated under vacuum for 10 min. Thereafter, the infiltrated material was centrifuged (200 *g* for 20 min at 4 °C) in swinging buckets (CTR429, Jouan centrifuge). The resulting fluids were collected at the bottom of the tubes. The processes of vacuum infiltration and centrifugation were repeated once. Finally, the pieces of culms were infiltrated again and centrifuged, as in the previous step, in a solution containing 2 M LiCl instead of CaCl_2_. The protein extracts were desalted on EconoPac® 10DG column (BIO-RAD, Hercules, CA, USA) as described [42]. Proteins were then solubilized in UHQ water and quantified by the CooAssay Protein Assay kit (Interchim, Montluçon, France) according to a modified Bradford method [78].

In order to verify the quality of the extractions, 40 μg of proteins were separated by 1D-electrophoresis as described [79]. After that, the staining of the bands was carried out with a Coomassie Brilliant Blue (CBB)-based method [80]. The image of the gel was obtained through a scanner (GE-III Image scanner, GE Healthcare, Ramonville Saint-Agne, France).

### MS^E^ analysis

Sample preparation was performed as described [28]. However, after increasing pH by adding 5 μL of 1 N NH_4_OH, an additional step was performed: the addition of phosphorylase B-rabbit (Waters, Manchester, UK) as an internal standard (2.5 μL of 1 pmol.μL^−1^) to the digested aliquot (80 μL). Consequently, 17.5 μL of 20 mM ammonium formate was added to the vials, reaching a final volume of 100 μL.

For each extract, 5 μL of the total protein digest (containing 3 μg of proteins) were fractionated by reverse-phase ultraperformance liquid chromatography (2D- nanoACQUITY UPLC®, Waters®, Manchester, UK). Separation in two dimensions, elution and trapping were performed as described [28]. Acquisition of MS data used a Synapt G2 HDMS equipped with an ion mobility cell and a NanoLockSpray source in the positive ion and ‘V’mode (Waters®), with the same parameters as described [28]. MS experiments were performed by switching between low (3 eV) and high collision energies (15–50 eV) applied to the ‘T-wave’ cell trap, filled with argon. The low and high energy scans from m/z 50 to 2000 used a scan time of 0.8 s. The intensities of the spectra were calculated using the stoichiometric method during MS experiments, according to the internal standard, to identify and quantify the proteins [81].

The doubly-charged ion ([M + 2H] ^2+^) was used for initial single-point calibration and MS/MS fragment ions of GFP [Glu 1]-Fibrinopeptide B *m/z* 785,84,206 ([M + 2H] ^2+^) (Waters, Corp., Milford, USA) were used as lock masses and instrument calibration. Data-independent scanning (MS^E^) experiments were performed by switching between low (3 eV) and elevated collision energies (15–50 eV), applied to the trap ‘T-wave’ cell filled with argon. Scan time of 0.8 s were used for low and high energy scans from *m/z* 50 to 2000 [81].

### Identification and annotation of proteins

The bioinformatics analysis was performed as described [28]. However, since phosphorylase B-rabbit (Waters) was used as an internal standard to quantify peptides in the present study, its sequence was added to the SUCEST-translated EST database. The quantification of the proteins, in femtomoles, was obtained as an average from the biological replicates. Proteins were noted as “identified” when quantification was not possible due to low abundance (Table 1). The PGLS 2.5.1 expression data values of *p < 0.05* and *p > 0.95* were considered as statistically significant for down or up-regulation, respectively, considering the quantitative protein ratio DT method/ND Method.

Two biological replicates were performed in this study, and only proteins presented in both replicates were considered. Proteins were considered to be secreted and named CWPs when it was possible to predict a signal peptide with at least two bioinformatics programs, when no intracellular retention signal was predicted and when no more than one trans-membrane domain was predicted [28]. This work was done either manually for sugarcane translated ESTs or using ProtAnnDB for *S. bicolor* sequences [82]. In order to find *S. bicolor* (the closest species with a fully sequenced genome) homologous genes for the identified sugarcane ESTs, a blastp search was performed [83], as described [28]. Only proteins showing at least one specific peptide were considered.

CWPs were distributed into eight functional classes according to their annotation using InterPro [84] and PFAM [85]. All the data have been included in the *WallProtDB* database [43].

The median of the quantified proteins identified was calculated, being considered as T1R3 and T2R3, for the DT and ND Methods, respectively. Statistical processing was performed with MetaboAnalyst software 2.0 [86]. The quantitative data were normalized by the median, followed by a logarithmic transformation (Log2) and Pareto Scaling. The Partial-Least Squares Discriminant Analysis (PLS-DA) was used for the data analysis. In PLS-DA, R^2^ values were observed, which indicate how much of the total variation in the dataset is described by the analysis components, and Q^2^ values, which indicate how accurately the model can predict class membership. Both of them, therefore, are performance indicators [87]. The PLS-DA models were constructed and the importance of the variable in the projection (VIP) was used to identify the 15 ions that had a higher discrimination between the groups in the component with the highest power projection.

Besides the multivariate approaches, the univariate method (Student’s t- test and fold change) was performed to measure the significance of each protein in distinguishing the DT and ND Methods groups. The fold change threshold (x 4) and t-tests threshold 0.05 were adopted. To assess whether the proteins highlighted in the loading scores were statistically significant, a Volcano analysis was performed.

### Availability of supporting data

The proteomics data have been included in the *WallProtDB* public database (http://www.polebio.lrsv.ups-tlse.fr/WallProtDB/).

## Additional files

Additional file 1:
**Raw MS data from sugarcane culms using Method 1, biological replicate1.** (XLS 2.26 MB) Additional file 2:
**Raw MS data from sugarcane culms using Method 1, biological replicate 2.** (XLS 3.02 MB) Additional file 3:
**Raw MS data from sugarcane culms using Method 2, biological replicate1.** (XLS 2.33 MB) Additional file 4:
**Raw MS data from sugarcane culms using Method 2, biological replicate2.** (XLS 1.39 MB) Additional file 5:
**CWPs identified in 2 month-old sugarcane culms.** (XLSX 30.3 kb) Additional file 6:
**Proteins of sugarcane young culms identified using Method 1 or 2 and predicted to be intracellular.** (XLSX 21.2 kb) Additional file 7:
**Important features identified by Volcano plot.** (XLSX 10.0 kb)

## Abbreviations

CWP: cell wall protein
DT: Destructive
EST: expressed sequenced tag
FC: fold change
GH: glycoside hydrolase
LRR: leucine-rich repeat
LTP: lipid transfer protein
MS: mass spectrometry
ND: non-destructive
PME: pectin methylesterase
Prx: class III peroxidase
UHQ: ultra high quality
UPLC: ultra Performance Liquid Chromatography
